# AKT signalling selectively regulates PINK1 mitophagy in SHSY5Y cells and human iPSC-derived neurons

**DOI:** 10.1038/s41598-018-26949-6

**Published:** 2018-06-11

**Authors:** Marc P. M. Soutar, Liam Kempthorne, Shuichi Miyakawa, Emily Annuario, Daniela Melandri, Jasmine Harley, Gregory A. O’Sullivan, Selina Wray, David C. Hancock, Mark R. Cookson, Julian Downward, Mark Carlton, Hélène Plun-Favreau

**Affiliations:** 1 Department of Molecular Neuroscience, UCL Institute of Neurology, Queen Square, London, WC1N 3BG UK; 2 Biomolecular Research Laboratories, Pharmaceutical Research Division, Takeda Pharmaceutical Company Limited, Fujisawa, Japan; 3 CereVance Ltd. 418 Science Park, Milton Rd, Cambridge, CB4 0PZ UK; 4 The Francis Crick Institute, 1 Midland Road, London, NW1 1AT UK; 5 Laboratory of Neurogenetics, NIH, Building 35, Room 1A116, 35 Convent Drive, Bethesda, MD 20814 USA

## Abstract

The discovery of mutations within genes associated with autosomal recessive Parkinson’s disease allowed for the identification of PINK1/Parkin regulated mitophagy as an important pathway for the removal of damaged mitochondria. While recent studies suggest that AKT-dependent signalling regulates Parkin recruitment to depolarised mitochondria, little is known as to whether this can also regulate PINK1 mitochondrial accumulation and downstream mitophagy. Here, we demonstrate that inhibition of AKT signalling decreases endogenous PINK1 accumulation in response to mitochondria depolarisation, subsequent Parkin recruitment, phosphorylation of ubiquitin, and ultimately mitophagy. Conversely, we show that upon stimulation of AKT signalling via insulin, the mitophagy pathway is increased in SHSY5Y cells. These data suggest that AKT signalling is an upstream regulator of PINK1 accumulation on damaged mitochondria. Importantly, we show that the AKT pathway also regulates endogenous PINK1-dependent mitophagy in human iPSC-derived neurons.

## Introduction

Mitophagy is a form of selective autophagy for the removal of damaged mitochondria. Increasing evidence suggests that mitophagy is a protective mechanism that avoids toxic accumulation of heavily damaged mitochondria within the cell, thus contributing to the maintenance of a healthy mitochondrial network^1,2^. Deficiencies in mitophagy can lead to oxidative damage and cell death^3^; therefore, it is unsurprising that abnormal regulation of mitophagy has been found to contribute to varying disease pathologies including neurodegenerative diseases such as Parkinson’s disease (PD)^4–7^.

Much of what we know about the mitophagy pathway is based on the investigation of genetic forms of PD, where mutations were found at recessive loci *PARK6* (PINK1 (PTEN-induced putative kinase 1)) and *PARK2* (Parkin)^8,9^. The mitochondrial kinase PINK1 and ubiquitin E3 ligase Parkin act in concert to regulate the mitophagy process^10,11^. PINK1 selectively accumulates on the outer membrane of damaged mitochondria, where it facilitates Parkin recruitment from the cytoplasm to the mitochondria and activates its E3 ligase activity causing ubiquitination of mitochondrial membrane proteins^12–20^. Mitochondrial ubiquitin is phosphorylated by PINK1 and phospho-ubiquitin chains act as receptors for adaptor proteins (e.g. p62), triggering the engulfment of damaged mitochondria in autophagosomes, and the ultimate fusion with lysosomes leading to degradation of the targeted mitochondria^21^.

Whilst a detailed characterisation of the mitophagy pathway downstream of PINK1 accumulation and Parkin recruitment has been achieved over the last decade, the physiological stimuli and upstream signalling pathway(s) that regulate these processes remain poorly understood.

AKT is a Serine/Threonine kinase involved in many cellular processes^22^, including the recruitment of Parkin to damaged mitochondria^23^. Here we show that selective AKT inhibition in SHSY5Y FLAG-Parkin over-expressing neuroblastoma cells attenuates PINK1 accumulation in response to mitochondria depolarisation. This effect correlates with reduced recruitment of FLAG-Parkin to damaged mitochondria, reduced ubiquitin phosphorylation and p62 recruitment, thus decreasing the clearance of depolarised mitochondria. We further corroborate these results using human induced pluripotent stem cell (iPSC) derived cortical neurons, using a newly established mitophagy induction protocol. Our results further strengthen the role of the PI3K/AKT pathway in modulating PINK1/Parkin-dependent mitophagy.

## Results

### AKT signalling regulates PINK1 accumulation and Parkin recruitment to depolarised mitochondria

**Figure 1:**
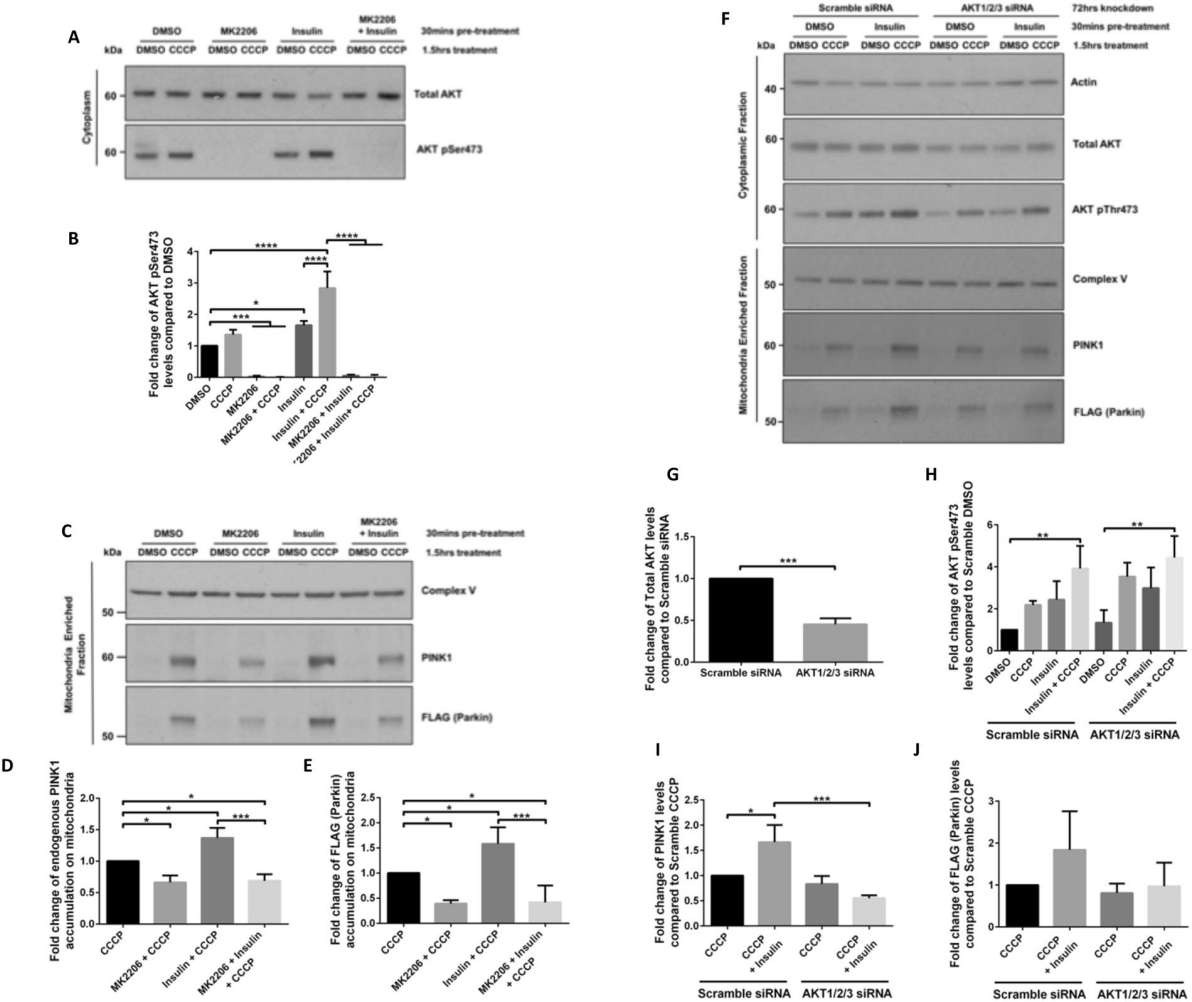
AKT signalling regulates PINK1 accumulation and FLAG-Parkin recruitment to depolarised mitochondria. (**A–E**). SHSY5Y cells expressing FLAG-Parkin were incubated 15 mins +/− 2.5 μM AKT inhibitor MK2206 prior to stimulation +/− 100 nM insulin for 15 mins. 10 μM CCCP was then added for 1.5 hrs to depolarise mitochondria and activate mitophagy. Cytoplasmic (**A,B**) and mitochondrial (**C–E**) enriched fractions were generated and samples were run on SDS-PAGE gels followed by WB using the indicated antibodies (cropped blots - full-length blots are presented in Supplementary Data). (**B**) graphs display quantified images showing fold changes in cytoplasmic AKT pSer473, while D and E graphs display quantified images showing fold changes in PINK1 accumulation and FLAG-Parkin (**C**) recruitment to mitochondria fractions (N = 3). (**F–J**). SHSY5Y cells expressing FLAG-Parkin cells were transfected with 5 nM individual siRNAs for AKT1, 2 and 3 for 72 hrs prior +/− 100 nM insulin for 15 mins. 10 μM CCCP was then added for 1.5 hrs to depolarise mitochondria and activate the mitophagy pathway. Cell lysates were then fractionated into cytoplasmic and mitochondria enriched samples (**F**) that were run on SDS-PAGE gels followed by WB using the indicated antibodies (cropped blots - full-length blots are presented in Supplementary Data). Figure 1 graphs display quantified images showing siRNA knockdown efficiency (**G**), fold changes in cytoplasmic AKT pSer473 (**H**), PINK1 accumulation (**I**) and FLAG-Parkin (**J**) recruitment to mitochondria fractions (N = 3).

While DJ-1, AKT and hexokinase activity were shown to regulate Parkin recruitment following mitochondrial depolarisation^23,24^, whether the AKT pathway modulates PINK1 accumulation and mitochondrial clearance remains unknown. SHSY5Y cells stably expressing FLAG-Parkin were pre-treated with either insulin, which stimulates AKT activity through activation of PI3K^25^, and/or MK2206, a potent allosteric inhibitor of AKT1, 2, and 3 activity^26^, prior to mitochondrial depolarisation with carbonyl cyanide m-chlorophenyl hydrazone (CCCP), which activates PINK1/Parkin-dependent mitophagy^27^. Mitochondrial and cytosolic enriched fractions were tested for their purity by Western blotting (WB), using ATPB (Complex V) and GAPDH markers, respectively (see Supplementary Fig. S1). WB of enriched cytoplasmic fractions demonstrated that insulin induced a robust phosphorylation of AKT at Ser473 in SHSY5Y FLAG-Parkin cells, which was blocked by MK2206 pre-treatment (Fig. 1A,B). CCCP treatments also stimulated AKT Ser473 phosphorylation. Pre-treatment of SHSY5Y FLAG-Parkin cells with MK2206 diminished endogenous PINK1 accumulation and FLAG-Parkin recruitment to the mitochondrial fraction in CCCP-treated cells, whilst insulin increased both. The insulin-mediated increase in endogenous PINK1 mitochondrial accumulation and FLAG-Parkin recruitment in CCCP treated cells was blocked when cells were pre-treated with MK2206 (Fig. 1C–E). These data were corroborated using a ATP competitive AKT inhibitor (GDC-0068) (see Supplementary Fig. S2A and B) and further confirmed using genetic ablation of AKT proteins. siRNA targeting AKT1/2/3 led to a ∼50% knockdown of total AKT levels (Fig. 1F,G), while the ratio of phospho-Ser473 and total AKT was similar in the AKT knockdown cells as compared to scramble control (Fig. 1F,H). Knockdown of AKT reduced endogenous PINK1 accumulation stimulated by insulin (Fig. 1F,I) and led a trend towards decreased FLAG-Parkin recruitment, although the trend didn’t reach statistical significance (Fig. 1F,J). These data further confirmed that AKT signalling regulates PINK1 accumulation and subsequent Parkin recruitment to depolarised mitochondria. On its own, AKT knockdown had no effect on PINK1 accumulation and Parkin recruitment following CCCP- treatment (Fig. 1F,I and J). This is very likely explained by the remaining 50% AKT protein levels in knockdown cells (Fig. 1G).

In order to determine whether the regulation of mitochondrial PINK1/Parkin redistribution was due to modulation of mitochondrial membrane potential (ΔΨm), tetramethylrhodamine methyl ester (TMRM) fluorescence was measured following treatment with MK2206 with or without insulin, prior to CCCP treatment (see Supplementary method). While mitochondria were hyperpolarised following MK2206 treatment, neither MK2206 nor insulin affected CCCP’s ability to dissipate ΔΨm (see Supplementary Fig. S3A and B), suggesting that MK2206-dependent inhibition of PINK1 accumulation and Parkin recruitment was not due to inhibition of CCCP-induced mitochondrial depolarisation.

AKT phosphorylation was previously shown to inhibit the function of Foxo3, a transcription factor regulating PINK1 mRNA expression^28–30^. In order to assess whether PINK1 mRNA levels were modulated following AKT inhibition, RT-PCR was performed in cells treated with CCCP and/or MK2206 (see Supplementary Fig. S4). These data revealed that CCCP treatment decreased PINK1 mRNA levels, while pre-treatment with MK2206 prevented this. These data confirm that AKT signalling can modulate PINK1 mRNA expression, likely via inhibited Foxo3-dependent as previously shown^29^, and further suggest that the MK2206 effect on CCCP-induced PINK1 accumulation is not due to its effect on PINK1 transcription, but rather regulates accumulation of PINK1 protein at the surface of depolarised mitochondria.

**Figure 2:**
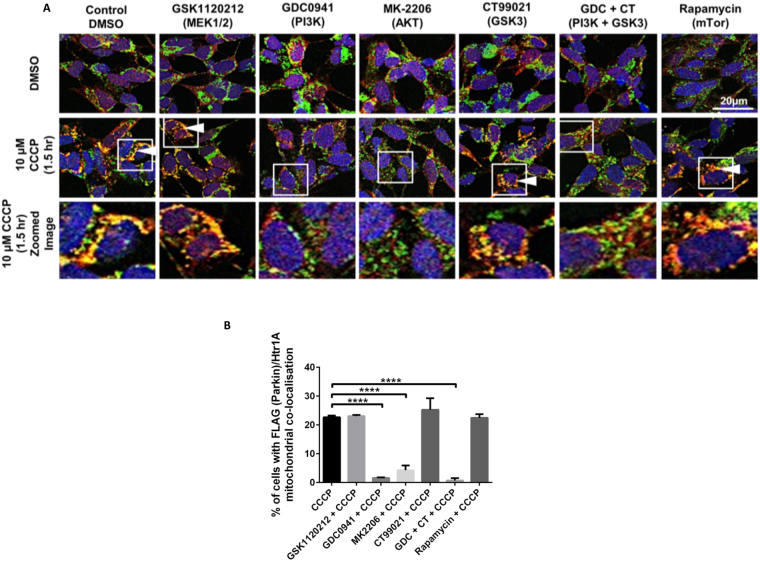
FLAG-Parkin recruitment to depolarised mitochondria is selectively regulated by AKT signalling. (**A,B**) SHSY5Y cells expressing FLAG-Parkin cells were pre-treated with compounds (see Table 1) for 30 minutes, +/− 100 nM insulin stimulation, prior to treatment with 10 μM CCCP for the times indicated in figure to depolarise the mitochondria. The cells were then fixed and prepared for immunocytochemistry. Confocal microscopy was used to capture images (Zeiss 710 VIS CLSM Green 488 nm = HtrA2, Red 568 nm = FLAG-Parkin, Blue = DAPI (nucleus)). Graphs display quantified images showing % cells with FLAG-Parkin colocalisation punctate with HtrA2 mitochondrial marker. White arrows indicate examples of cells with FLAG-Parkin and mitochondria colocalisation (N = 3).

AKT inhibition does not completely inhibit endogenous PINK1 accumulation or FLAG-Parkin recruitment (Fig. 1C–E), suggesting that additional signalling pathways may regulate PINK1/Parkin-dependent mitophagy. A number of pharmacological compounds (see Table 1) targeting kinases proposed to regulate energy sensing pathways, mitochondria function, or autophagy^31–34^ were assessed for their ability to modulate FLAG-Parkin recruitment to CCCP-treated SHSY5Y cells (see Supplementary Fig. S5A–C for WBs demonstrating kinase inhibitor efficacy). Immunocytochemistry (ICC) was used to assess FLAG-Parkin recruitment to mitochondria, using the mitochondrial marker HtrA2^35,36^. FLAG-Parkin recruitment to CCCP-depolarised mitochondria was decreased upon AKT and PI3K inhibition, an upstream regulator of AKT (Fig. 2A,B), when compared to CCCP alone, corroborating data shown in Fig. 1. Furthermore, these data confirmed that modulation of FLAG-Parkin recruitment to CCCP-treated mitochondria was specific to the PI3K/AKT pathway as inhibition of mTORC1, MEK1/2, or GSK3 α/β kinases had no effect (Fig. 2A,B).

**Table 1:** Information on kinase inhibitors.

Kinase	Inhibitor	Working Concentration	Isoform Inhibition
MEK1/2	GSK1120212	50 nM	MEK1, MEK2
PI3K	GDC0941	1 μM	P110 α, β, γ; DN A-PK; mTOR
AKT	MK2206	2.5 μM	AKT1, AKT2, AKT3
GSK3	CT99021	2 μM	GSK3 α, β
mTOR	Rapamycin	100 nM	mTOR Complex 1

AKT inhibition does not completely inhibit endogenous PINK1 accumulation or FLAG-Parkin recruitment (Fig. 1A–C), suggesting that additional signalling pathways may regulate PINK1/Parkin-dependent mitophagy. A number of pharmacological compounds (see Supplementary Fig. S5A–C and Table 1) targeting kinases proposed to regulate energy sensing pathways, mitochondria function, or autophagy^31–34^ were assessed for their ability to modulate FLAG-Parkin recruitment to CCCP-treated SHSY5Y cells (see Supplementary Fig. S5 for WBs demonstrating kinase inhibitor efficacy). Immunocytochemistry (ICC) was used to assess FLAG-Parkin recruitment to mitochondria, via assessing co-localisation with mitochondrial marker HtrA2^35,36^. FLAG-Parkin recruitment to CCCP-depolarised mitochondria was decreased upon AKT and PI3K inhibition, an upstream regulator of AKT (Fig. 2A/B), when compared to CCCP alone, corroborating data shown in Fig. 1. Furthermore, these data confirm that modulation of FLAG-Parkin recruitment to CCCP-treated mitochondria is specific to the PI3K/AKT pathway as inhibition of mTORC1, MEK1/2, or GSK3 α/β had no effect (Fig. 2A,B).

### AKT signalling regulates phosphorylation of ubiquitin, p62 accumulation and clearance of depolarised mitochondria

**Figure 3:**
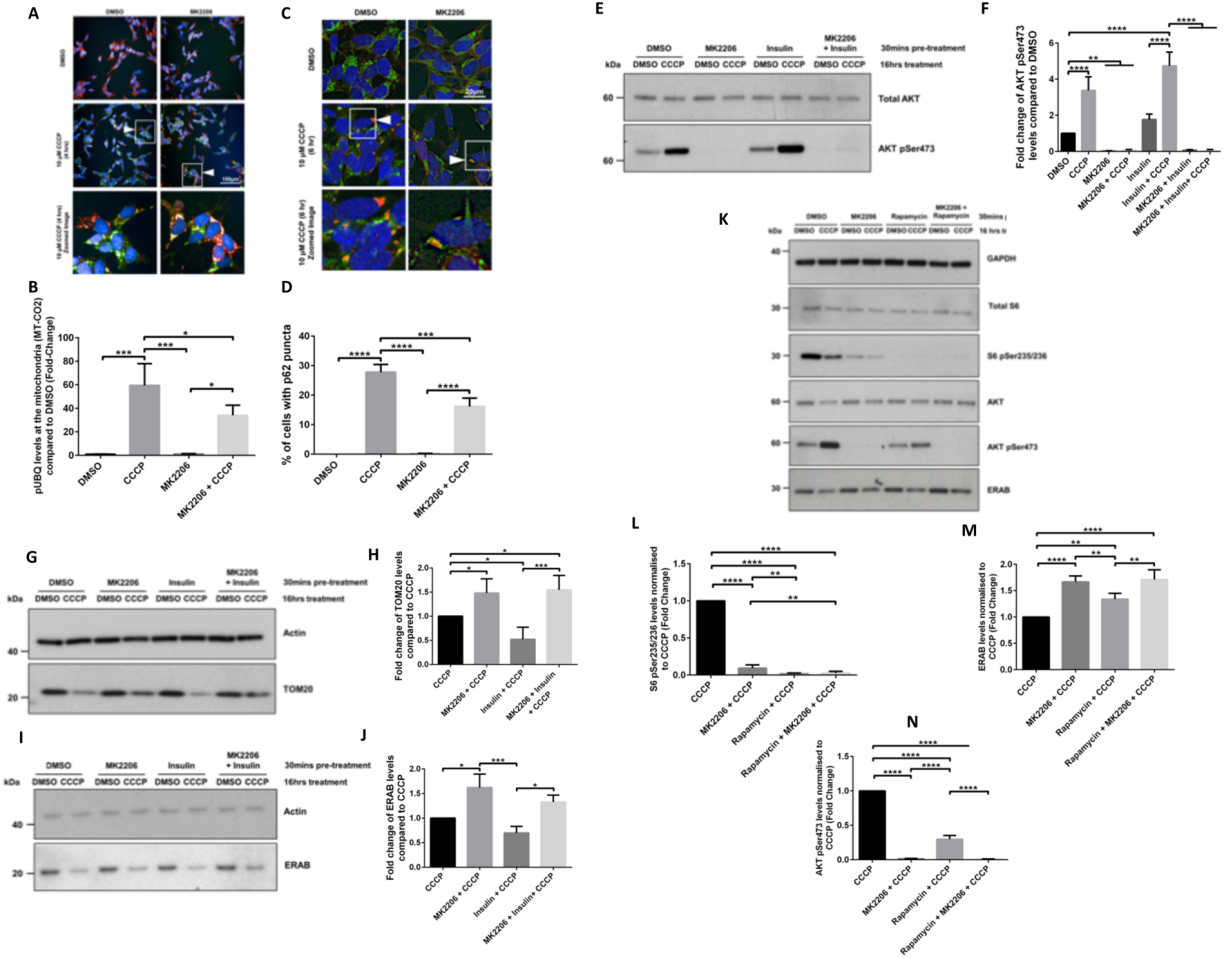
AKT signalling contributes to the regulation of mitophagy. (**A–D**) SHSY5Y cells expressing FLAG-Parkin cells were treated +/− AKT inhibitor MK2206 (2.5 μM for 15 min) prior to +/− 100 nM insulin stimulation for 15 mins. Subsequently, 10 μM CCCP was added for the indicated times and the cells were fixed and prepared for immunocytochemistry. (**A,B)**. Confocal microscopy (Opera Phenix^TM^) was used to capture UBQ pSer65 (Green 448 nm) and COXII (mitochondria Red 568 nm) images. White arrows indicate examples of cells with UBQ pSer65 and mitochondria (N = 3). (**C,D**). Confocal microscopy (Zeiss 710 VIS CLSM) was used to capture p62 (Red 568 nm) and TOM20 (mitochondria Green = 448 nm) images. White arrows indicate examples of cells with p62 and mitochondria (N = 3). (**E–J**). SHSY5Y cells expressing FLAG-Parkin cells were treated +/− AKT inhibitor MK2206 (2.5 μM for 15 min) prior to +/− 100 nM insulin stimulation for 15 mins. Subsequently, 10 μM CCCP was added for 16 hrs to depolarise mitochondria and activate the mitophagy pathway. In (**E,G,I**) whole cell lysates were run on SDS-PAGE gels and immunoblotted using the indicated antibodies (cropped blots - full-length blots are presented in Supplementary Data). (**F,H,J**) graphs display quantified images showing fold-change versus DMSO control (N = 4). (**K–N**). SHSY5Y cells expressing FLAG-Parkin were treated +/− AKT inhibitor MK2206 (2.5 μM for 15 min) and/or 100 nM Rapamycin for 30 mins. Subsequently, 10 μM CCCP was added for 16 hrs to depolarise mitochondria and activate the mitophagy pathway. In (**K**), whole cell lysates were run on SDS-PAGE gels and immunoblotted using the indicated antibodies (cropped blots - full-length blots are presented in Supplementary Data). (**L–N**) graphs display quantified images showing fold-change versus CCCP control (N = 4).

PINK1 has been shown to phosphorylate ubiquitin (UBQ) at Ser65 *in vitro* and in cells following mitochondrial depolarisation^12–19^. We therefore sought to determine whether MK2206-mediated inhibition of AKT kinase activity would regulate UBQ pSer65 levels in CCCP-treated cells (Fig. 3A,B). Using ICC, we showed that MK2206 pre-treatment reduced the levels of mitochondrial UBQ pSer65 in response to CCCP treatment, which was in keeping with reduced PINK1 mitochondrial accumulation under these conditions (Fig. 1), and corroborated data shown previously^24^. We further assessed MK2206’s effect on accumulation of the autophagosome cargo protein p62^37^. p62 puncta formation was assessed after 6 hrs CCCP treatment, and MK2206 treatment led to the % reduction of cells with p62 accumulation (Fig. 3C,D). Collectively these data further suggest that AKT kinase activity regulates PINK1-dependent ubiquitin phosphorylation and recruitment of the autophagy machinery.

In order to further assess whether the AKT signalling pathway regulates mitophagy, mitochondrial clearance was assessed 16 hrs after CCCP treatment. SHSY5Y cells stably expressing FLAG-Parkin were pre-treated with MK2206 and/or insulin for 30 mins prior to the addition of CCCP (Fig. 3E–H). Figure 3E,F shows that similar to short CCCP treatment (Fig. 1A,B), long CCCP treatment increased AKT Ser473 phosphorylation, and that this phosphorylation was further increased upon insulin stimulation, while MK2206 abolished it. Cells pre-treated with MK2206, prior to treatment with CCCP, showed higher levels of mitochondrial markers TOM20 (translocase of outer mitochondrial membrane 20) (Fig. 3G,H) and ERAB (mitochondria matrix) (Fig. 3I,J) than CCCP alone, confirming that AKT inhibition reduced mitophagy. Conversely, cells treated with insulin prior to CCCP had a higher level of mitophagy as compared to CCCP alone, as indicated by the lower levels of TOM20 and ERAB, (Fig. 3C–F), whilst MK2206 attenuated the effect of insulin on mitophagy.

Rapamycin, a mTOR inhibitor, has been shown to promote macroautophagy^38^. However, in our experiments, short term inhibition of mTOR activity with rapamycin did not affect FLAG-Parkin translocation to depolarised mitochondria (Fig. 2A,B). In order to determine whether long rapamycin treatment could counteract MK2206-mediated mitophagy inhibition, SHSY5Y FLAG-Parkin cells were treated with rapamycin for 16 hrs, leading to inhibition of mTOR activity, as indicated by loss of S6 Ser235/236 phosphorylation^39^ (Fig. 3K,L). While 16 hrs rapamycin treatment reduced CCCP-induced mitophagy (as indicated by the degradation of the mitochondria matrix marker ERAB), this was not as efficient as long MK2206 treatment (Fig. 3M). This could be due to the fact that long term treatment of cells with rapamycin has been shown to inhibit mTORC2, a kinase proposed to phosphorylate AKT at Ser473^40–42^. In fact, in our experiments, 16 hrs treatment with rapamycin did not completely inhibit CCCP-induced AKT Ser473 phosphorylation, on the contrary to MK2206 (Fig. 3K,N). Altogether, our data show that AKT activity modulates mitophagy, upstream of PINK1^43^.

### AKT signaling regulates mitophagy in human iPSC-derived cortical neurons

**Figure 4:**
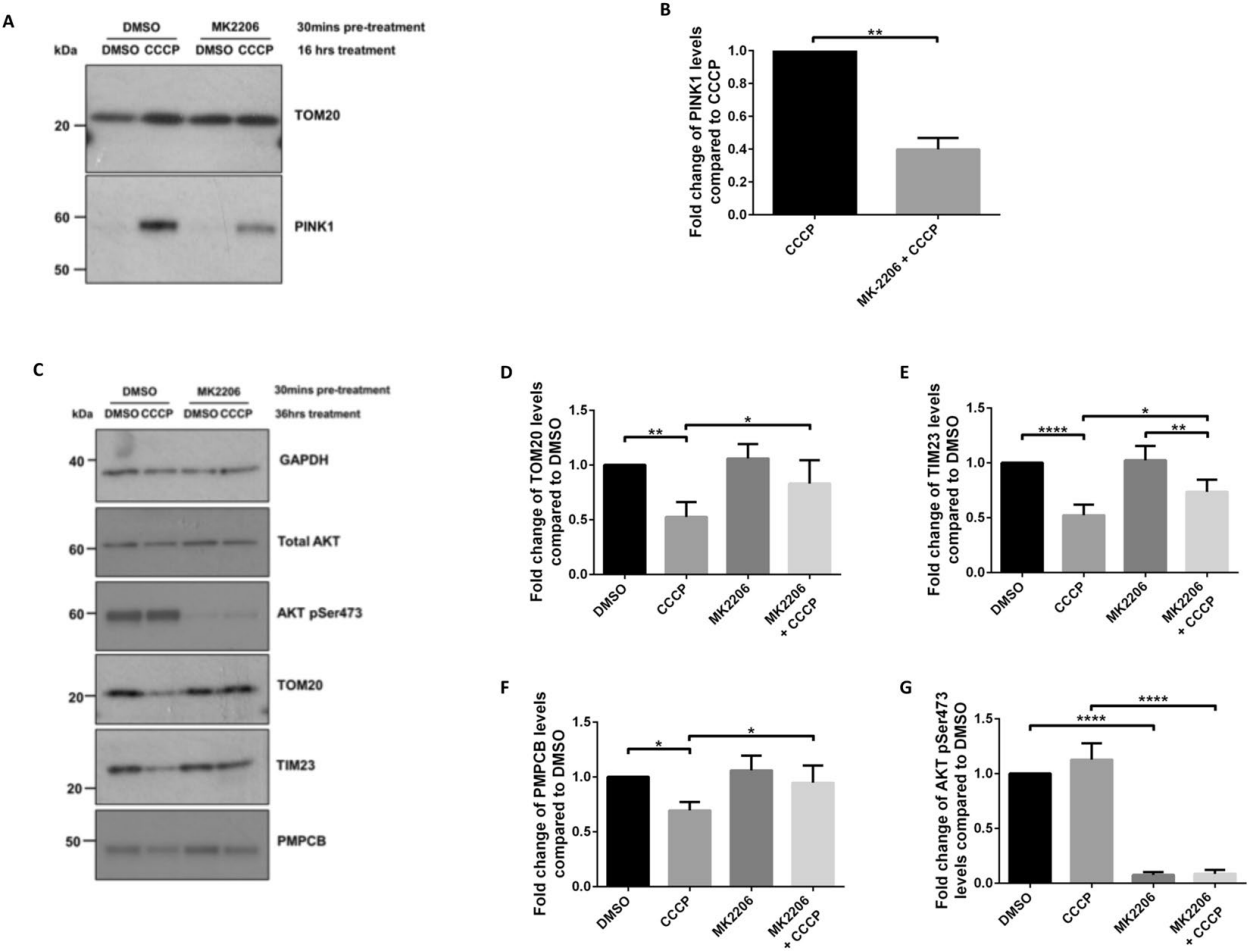
AKT kinase activity is required for mitophagy in iPSC cortical neurons. (**A,B**) Day 80 human iPSC-derived cortical neurons were treated +/− AKT inhibitor MK2206 (2.5 μM) for 30 mins prior to exposure to 10 μM CCCP to depolarise mitochondria and activate the mitophagy pathway. Human iPSC-derived cortical neurons were treated for 16 hrs, cell lysates were fractionated into mitochondria and cytoplasmic enriched samples that were run on SDS-PAGE gels and immunoblotted using the indicated antibodies (cropped blots - full-length blots are presented in Supplementary Data). In (**B**), the graph displays quantified images showing fold changes in PINK1 versus TOM20 loading control. Un-Paired Two-tailed T-test with Welch’s correction (N = 3) **p = 0.0044. (**C–E**) Human iPSC-derived cortical neurons were treated for 36 hrs with 10 μM CCCP before the whole cell lysates were run on SDS-PAGE gels and immunoblotted using the indicated antibodies (cropped blots - full-length blots are presented in Supplementary Data). (**D–G**) Graphs display quantified images showing fold-change versus DMSO control of TOM20, TIM23, PMPCB and pAKT Ser473, versus GAPDH loading control (N = 4).

Next, we determined whether AKT-mediated regulatison of mitophagy observed in the immortalised SHSY5Y cell line could be replicated in human iPSC-derived neurons expressing endogenous levels of PINK1 and Parkin. iPSC cortical neurons were pre-treated with MK2206 for 30 mins prior to 16 hrs of CCCP treatment (Fig. 4). Endogenous PINK1 accumulation was detected in mitochondrial fractions after 16 hrs treatment with CCCP, and MK2206 reduced the effect of CCCP (Fig. 4A,B). MK2206 alone had no effect on PINK1 accumulation, suggesting that AKT kinase activity regulates endogenous PINK1 levels in response to mitochondria depolarisation. Whether this effect translates into a reduction in mitochondrial protein degradation was further assessed using a new protocol for mitophagy induction in human iPSC-derived cortical neurons (see methods). Following 36 hrs of CCCP treatment, levels of outer and inner mitochondrial membrane markers TOM20 and TIM23 (translocase of inner mitochondrial membrane 23), respectively, and matrix marker PMPCB (Peptidase, Mitochondrial Processing Beta Subunit - mitochondria matrix protein) were reduced, as compared to DMSO control, showing that mitochondrial clearance is induced. Pre-treatment with MK2206 attenuated the effect of CCCP on TOM20, TIM23 and PMPCB (Fig. 4C–G), confirming that AKT signalling regulates mitophagy. MK2206 treatment reduced AKT pSer473 to almost undetectable levels, similar to what was observed in SHSY5Y cells. All together these data demonstrate that AKT signalling contributes to the regulation of PINK1 mitophagy in human iPSC-derived cortical neurons.

## Discussion

In this study, we show that AKT signalling regulates endogenous PINK1 accumulation, and subsequent efficient clearance of damaged mitochondria in SH-SY5Y cells and iPSC neurons.

Previous studies have shown that AKT and PINK1 regulate Reactive Oxidative Species (ROS) as well as glucose metabolism, mitochondria respiration, and apoptosis, all of which are associated with mitochondrial homeostasis (reviewed in^32^). Here we show that AKT and PINK1 act within a common biochemical pathway. Whether the regulatory effect of AKT signalling on PINK1 and Parkin is direct, or indirect (e.g. via modulation of ROS levels/oxidative stress pathways, via cellular/mitochondria metabolism, or via transcription of AKT/mitophagy related genes (e.g. HIF^44,45^)), is yet to be determined. Previous studies suggest that AKT inhibtion of FOXO3 reduces PINK1 mRNA levels^29^. Here we show that CCCP treatment increases AKT phosphorylation, while PINK1 mRNA levels are decreased, and this is inhibited by subsequent AKT inhibition via MK2206.While these data confirmed previous studies demonstrating that AKT signaling can modulate PINK1 mRNA expression, potentially via inhibited Foxo3-dependent^29^, the data presented throughout show that AKT can also regulate CCCP-induced PINK1 protein accumulation. We have also shown that CCCP treatment increases pSer473-AKT, which is further increased with insulin pre-treatment, as reported previously^46^. Other studies have shown that insulin-treated embryonic fibroblasts from PINK1-deficient mice, or PINK1-deficient T-cells show a significant reduction of pSer473-AKT^47,48^. Conversely, the over-expression of PINK1 increases AKT pSer473-AKT via mammalian target of rapamycin complex 2 (mTORC2), which is independent of PI3K^49,50^. While these studies suggest that PINK1 is upstream of AKT in the pathway, our study and others^23^ suggest that PINK1 is downstream of AKT. All together these data suggest that the AKT-PINK1 signalling pathways involve complex feedback loop mechanisms. Acute treatments of rapamycin have been shown to directly inhibit mTORC1, while mTORC2 is reported to be inhibited only after prolonged treatments^40^. In our study, rapamycin had no significant effect on FLAG-Parkin recruitment to mitochondria after CCCP treatment for 1.5 hrs, while mitophagy was decreased after 16 hrs rapamycin/CCCP treatment, even if not as efficiently as MK2206-mediated AKT inhibition. 16 hrs rapamycin treatment completely inhibited mTOR activity (as indicated by loss of pS6), as opposed to AKT phosphorylation. Our data further suggest interactions between mTORC2, AKT and PINK1 signalling.

Mitophagy plays an important role in neurodegenerative diseases^28,51–55^. Thus, monitoring mitophagy in neurons is essential. However, this has proven challenging and consequently the vast majority of studies have used immortalised cell lines, despite the limitations when interpreting the results in terms of relevance to neurons. Amongst other factors, high concentrations of mitochondrial depolarising agents, long time points and apoptotic inhibitors (reviewed in^56^) have been required to induce mitophagy in primary neuronal cultures over-expressing Parkin. As well as the lack of suitable cellular models and methods to activate mitophagy, the reagents available to detect endogenous PINK1 were a limiting factor and studies usually required non-physiological over-expression of Parkin. We have developed a new protocol to detect endogenous PINK1 accumulation and clearance of mitochondrial proteins in human iPSC-derived cortical neurons in response to CCCP treatment. Importantly, the AKT kinase activity dependent regulation of PINK1 accumulation and mitophagy observed in SHSY5Y cells was recapitulated in iPSC-derived neurons. Human tissue studies have shown that both total AKT and pSer473-AKT levels are low in tyrosine hydroxylase positive dopaminergic neurons from PD patient brains relative to pathologically normal control brains^57^. Moreover, compounds that activate AKT kinase activity, or AKT overexpression have been shown to be neuroprotective in toxin-induced PD models^58,59^. Whether the AKT dependent regulation of PINK1 accumulation to damaged mitochondria and subsequent mitophagy may be part of this mechanism remains to be determined.

Improving our understanding of the mitophagy process will have benefits outside PINK1/Parkin PD research e.g. the known link between mitochondria dysfunction and diabetes^60–64^. It is noteworthy that Type 2 diabetes has been associated with an increased risk of developing PD via an unknown mechanism^65^. Interestingly, diabetes patients display deficits in insulin signalling that is likely to be related to observed reduced AKT signalling^66^. It may therefore be important to study mitophagy in the context of insulin resistance.

Mitochondria dysfunction and defective mitophagy has been shown to be important features of several neurodegenerative diseases such as PD, Alzheimer’s diseases, Huntington’s disease and Amyotrophic Lateral Sclerosis^28,51–55^. Thus, a better understanding of mitochondrial quality control could allow for new opportunities for therapeutic intervention associated with these non-curable diseases.

## Methods

### Chemicals and antibodies

Unless otherwise stated, all tissue culture reagents were obtained from Life Technologies, Paisley, U.K. and standard chemicals were obtained from Sigma-Aldrich, Dorset, U.K.

Unless otherwise indicated, all antibodies were purchased from Cell Signalling (Leiden, Netherlands), apart from anti-mouse FLAG (M2; F3165, Sigma-Aldrich, U.K.), rabbit anti-FLAG (Sigma-Aldrich, Dorset, U.K.), mouse anti-ATPB (Complex V) and MTCO2 (Abcam, Cambridge, U.K.), anti-CRMP2 (Protein Phosphate Unit, University of Dundee, U.K.), pThr509 CRMP2 (St Johns Laboratory) TIM23 and p62 (BD Biosciences, Oxford, U.K.), TOM20 (Santa Cruz Biotechnology, Heidelberg, Germany), PMPCB (Proteintech, Manchester, UK)

The rabbit monoclonal antibody (mAb) against human PINK1 was generated using a modified version of a previously described method^67^. Western blotting of lysates from SHSY5Y transfected with PINK1 siRNA was used to test the specificity of the PINK1 mAb antibody (see Supplementary Fig. S6). PINK1 and AKT1/2/3 siGenome SMART pool SiRNA and Dharmafect transfection reagent was purchased from Dharmacon, Horizon Discovery Ltd, 8100 Cambridge Research Park, Waterbeach, Cambridge, UK.

### SHSY5Y Cell culture

SHSY5Y neuroblastoma cells overexpressing FLAG-Parkin were a kind gift from H. Ardley (Institute of Molecular Medicine, Leeds, U.K.) and the methods for its generation has been described elsewhere^68^. These cells were cultured in Dulbecco’s modified Eagle medium (DMEM) containing 4.5 g/L glucose and supplemented with 10% heat-inactivated foetal bovine serum (FBS) in a humidified chamber at 37 °C with 5% CO_2_. Where indicated, cells were treated with 10 μM CCCP for the indicated times before harvesting lysates for Western blotting or fixing for immunostaining.

### SiRNA knockdown

Transient Scramble or PINK1 or AKT1/2/3 knockdown in SHSY5Y FLAG-Parkin expressing cells were generated by transfecting either non-targeting scramble siRNA or a pool of four siRNA constructs targeting human protein (siGenome SMARTpool) using Dharmafect transfection reagent. Cells were transfected with their respective SiRNAs for 72 hrs prior to treatments.

### Induced pluripotent stem cell (iPSC)-derived cortical neuron culture

Induced pluripotent stem cells (iPSCs) were cultured on Geltrex basement membrane matrix and maintained in Essential 8 media. Dual SMAD inhibition was used to induce cortical neurogenesis, as described previously^69^. Briefly, iPSCs were grown to 100% confluence before addition of neural induction media containing N-2 (DMEM/F-12 GlutaMAX, N-2 supplement, 5 μg/ml insulin, 1 mM L-glutamine, 100 μl nonessential amino acids, 100 M 2-mercaptoethanol, 50 U/ml penicillin and 50 mg/ml streptomycin) and B-27 (B-27 medium consists of Neurobasal, B-27 supplement, 200 mM L-glutamine, 50 U/ml penicillin and 50 mg/ml streptomycin) medias in a 1:1 ratio supplemented with the SMAD inhibitors (1 μM dorsomorphin (Tocris, Bristol, U.K.) and 10 μM SB431452 (Tocris, Bristol, U.K.)). The media was changed on a daily basis for 10 days during which iPSCs convert into a neuroepithelial layer. At day 10, dispase was used to lift the neuroepithelial layer that was then replated onto laminin-coated plates. Cells were subsequently maintained with neural maintenance media containing 1:1 ratio of N2 and B27 media. Once considerable neurogenesis has occurred, cells were split using accutase. For the final plating, cells were grown on Geltrex-coated plates.

### RNA processing and analysis

Treated SHSY5Y (FLAG-Parkin overexpressing) cells were lysed in TRIzol^TM^ (Invitrogen, California, USA) and RNA was isolated according to manufacturer’s protocol. Isolated RNA was quantified and using NanoDrop 3300 (ThermoFisher, Massachusetts, USA). Approximately 1 µg of RNA was reverse transcribed following DNase treatment using SuperScript® IV Reverse Transcriptase kit (Invitrogen). Control samples were generated following the same protocol without the addition of Reverse Transcriptase. RNA levels were analysed via PCR using SYBR Power-Up Master Mix (ThermoFisher, Massachusetts, USA) and the QuantStudio Flex system (Applied Biosystems, ThermoFisher, USA). PINK1 RNA levels were assessed Forward: 5′-GTGGAACATCTCGGCAGGTT-3′ and Reverse: 5′-CCTCTCTTGGATTTTCTGTAAGTGAC-3′ and double-normalised to GAPDH (primers: forward ATGACATCAAGAAGGTGGTG; reverse CATACCAGGAATGAGCTTG) and MRPL19 (primers: forward GGGATTTGCATTCAGAGATCAG; reverse GGAAGGGCATCTCGTAAG) house-keeping genes. House-keeping genes were also normalised against each other revealing a linear relationship between samples. Data was double-normalized to both house-keeping genes and displayed as fold-change compared to control (DMSO treatment) using the 2^−∆∆Ct^ method. Experiments were performed on biological N = 2 and technical N = 3 for each biological repeat.

### Induction of mitophagy in iPSC-derived cortical neurons

The neural maintenance medium on human iPSC-derived cortical neurons (80 days post induction) was replaced 1 hr before treatment with experimental medium (N-2 (DMEM/F-12 GlutaMAX, N-2 supplement, 5 μg/ml insulin, 1 mM L-glutamine, 100 μl nonessential amino acids, 50 U/ml penicillin and 50 mg/ml streptomycin) and B-27 (Neurobasal, B-27 No antioxidant supplement, 200 mM L-glutamine, 50 U/ml penicillin and 50 mg/ml streptomycin) media in a 1:1 ratio. Every 12 hrs, 50% of the media was removed and replaced with fresh media containing 10 μM CCCP (used to initiate mitophagy) and/or treatments, for the indicated times, whereby cell lysates were created for Western blotting or cells fixed for immunostaining.

### Mitochondria isolation by centrifugation

Post-treated cells were washed with PBS before addition of mitochondrial homogenisation buffer (250 mM sucrose, 1 mM EDTA (Edetate disodium salt dehydrate), 10 mM Tris, pH 7.4 supplemented with protease and phosphatase inhibitors). Cell plates were then frozen at −80 °C overnight. The following morning, plates were thawed on ice then lysates scraped into 1.5 ml centrifuged tubes, before a 20x trituration. Sample were then frozen at −80 °C for 1 hr. Cell lysates were thawed on ice before cell debris and non-lysed cells removed by centrifugation at 1,500 g for 10 minutes. Subsequently, the supernatant was centrifuged at 12,000 g for 10 minutes to pellet the mitochondria, followed by 2 washes with homogenisation buffer to remove any cytoplasmic contamination. Isolated mitochondria pellets were suspended in 1x NuPAGE® LDS sample buffer supplemented with 10 mM dithiothreitol (DTT) prior to SDS-PAGE and Western blotting, then heated to 70 °C for 10 mins.

### Immunoblotting and Immunofluorescence

SDS-PAGE and Western blotting methods are detailed elsewhere^70^. Cropped gels were used to improve presentation clarity. Full-length blots are presented in the Supplementary figures^71^.

For immunofluorescence experiments, the cells were fixed with 4% (w/v) formaldehyde/PBS solution for 5 min at room temperature before permeabilisation using a 0.5% (v/v) Triton X-100/PBS solution for 15 min. Samples were blocked for 30 min in 10% (v/v) FBS/PBS/0.5% (v/v) Triton X-100 solution before addition of the primary antibody for 2 hr in blocking solution. The appropriate secondary antibodies (AlexaFluor 488 and 568 secondary species-specific antibodies) were added in blocking solution for 1 hr. Cells on glass slides were mounted using ProLong® Gold Antifade Reagent with DAPI (cell nuclei staining) (Life Technologies, Paisley, U.K.), while cells retained in cell culture plates were given a 5 min DAPI PBS wash and maintained in PBS before measuring.

### Image Processing

Scanned Western blot images were processed before the bands were quantified using ImageJ. For colocalisation studies images were captured on a Zeiss 710 VIS CLSM confocal microscope (Zeiss GmbH, Jena, Germany) using a META detection system. To quantify the accumulation of FLAG-Parkin or p62 markers at the mitochondria (as demarcated using HtrA2 or TOM20), one independently non-blinded and 2 blinded observers visually scored cells using Volocity (Perkin Elmer). Cells with no marker signal co-localised with mitochondria was classified as a ‘no marker translocation’ cell. In order to score positively for mitochondrial accumulation, a cell was required to exhibit marker localisation with mitochondria and visualised as an overlaid channel. For each experiment, a minimum of 80 cells per field, at 3 different locations across 3 individual coverslips/wells was analysed.

For UBQ pSer65 colocalisation studies, images were captured on an Opera Phenix^TM^ High Content Screening confocal microscope (PerkinElemer Inc.) and the Columbus 2.8 analysis software (PerkinElemer Inc.) was used to quantify the amount of UBQ pSer65 accumulation at the mitochondria. A mitochondrial marker mask was created using MTCO2 (568 nm) where then only UBQ pSer65 signal (488 nm) was measured within those areas. Integrated intensity of spots was calculated by multiplying spot intensity by the area of mitochondria covered by the UBQ pSer65.

### Statistical Analysis

Independent experiment numbers (N number) are indicated in figure legends for each dataset. Data was subjected to One-way ANOVA with Holm-Sidak post-hoc analysis for pairwise comparison. For comparison of two data sets, Unpaired two-tailed T-test with Welch’s correction was performed. Error bars on all graphs indicate mean ± standard deviation from replicate experiments. In each instance *p < 0.05; **p < 0.01; ***p < 0.001; ****p < 0.0001; no * represents no significance. All statistical analyses and graph production was carried out using GraphPad Prism (version 6, GraphPad Software, La Jolla California USA).

### Data availability

All data generated or analysed during this study are included in this published article (and its Supplementary Information files).

## Electronic supplementary material

Supplementary Information
