# Supplementary material for: AKT signalling selectively regulates PINK1 mitophagy in SHSY5Y cells and human iPSC-derived neurons
Source: Sci Rep. 2018 Jun 11;8:8855. doi: 10.1038/s41598-018-26949-6 (PMC5995958; doi:10.1038/s41598-018-26949-6)

AKT signalling selectively regulates PINK1 mitophagy in SHSY5Y cells and human iPSC-derived neurons

Marc P.M. Soutar[1*], Liam Kempthorne[1], Shuichi Miyakawa[2], Emily Annuario[1], Daniela Melandri[1], Jasmine Harley[1], Gregory A. O'Sullivan[3], Selina Wray[1], David C. Hancock[4], Mark R. Cookson[5], Julian Downward[4], Mark Carlton[3], Hélène Plun-Favreau[1*]

Supplementary Information

Supplementary Methods

Supplementary Method Measurement of Mitochondrial Membrane Potential.
$\Delta\Psi m$ was estimated by live cell imaging using tetramethylrhodamine methyl ester (TMRM). This is a cationic, red-orange fluorescent dye that is sequestered by intact active mitochondria. SHSY5Y cells expressing FLAG-Parkin cells were loaded with 25 nM TMRM for 30 min 37 ºC in DMEM buffered with 10 mM HEPES prior to treatments. TMRM excitation wavelength is 560 nm and the emitted fluorescence was measured at 580 nm using Opera Phenix[TM] high content screening confocal microscope (Perkin Elmer Inc.). In order to analyse the treatment response, the Z-stack images were taken before and after treatment, where a reduction in TMRM fluorescence represents dissipation of $\Delta\Psi m$.

Supplementary Figure legends

Supplementary Figure S1. Demonstration of mitochondria enrichment efficiency.
Mitochondria fractions were produced from SHSY5Y cells expressing FLAG-Parkin. Representative mitochondrial enriched or cytoplasmic fractions were run on SDS-PAGE gels and immunoblotted using the indicated antibodies. GAPDH represents the cytoplasm whilst Complex ATPB the mitochondria.

Supplementary Figure S2. GDC-0068 AKT inhibitor inhibits PINK1 accumulation and Parkin recruitment to depolarised mitochondria.
SHSY5Y cells expressing FLAG-Parkin cells were incubated 15 mins +/- AKT inhibitor GDC-0068 prior to stimulation +/- 100 nM insulin for 15 mins. 10 µM CCCP was then added for 1.5 hrs to depolarise mitochondria and activate mitophagy. Cell lysates were fractionated into cytoplasmic and mitochondria enriched samples that were run on SDS-PAGE gels followed by WB using the indicated antibodies. The graph displays quantified images showing fold changes in PINK1 accumulation and FLAG-Parkin recruitment to mitochondria fractions (N=1). The inhibition of AKT kinase activity increases the activity of GSK3 α/β thus increasing CRMP pThr509 [69]. Thus, CRMP pThr509 was used as an indicator of AKT kinase activity.

Supplementary Figure S3. MK2206 doesn't prevent CCCP-induced mitochondrial depolarisation.
SHSY5Y cells expressing FLAG-Parkin were pre-treated with 2.5 µM MK2206, DMSO, and/or 100nM insulin 30 min prior to treatment with either 10 µM CCCP or DMSO. TMRM fluorescence mas measured after 30 min. TMRM total signal area per cell multiplied by the mean spot intensity gave the total cell load of TMRM expressed as TMRM levels. Mean TMRM levels are shown as normalised to DMSO treatments +/- S.D (N=3). Representative images of TMRM signal after 30 min exposure to 10 µM CCCP or DMSO with 2.5 µM MK2206, DMSO, and/or 100 nM insulin pre-treatments (Supplementary Fig. S3A).

Quantification is shown in Supplementary Fig. S3B.

Supplementary Figure S4. MK2206 inhibits CCCP-mediated decrease of PINK1 RNA levels.
SHSY5Y cells expressing FLAG-Parkin were incubated +/- 2.5 µM MK2206 for 15 mins prior to 10 µM CCCP treatment for 1.5 hrs. RNA was then extracted from these cells and PINK1 RNA levels were analysed after normalising to GAPDH and MRP19L (mitochondria ribosomal protein). Data displayed as fold-change ($2^{-\Delta\Delta C_t}$) compared to DMSO treatment (N=2).

Supplementary Figure S5. Kinase inhibitor efficacy.
SHSY5Y FLAG-Parkin cells were treated +/- inhibitor 30 mins prior to the addition of 10 µM CCCP for 1.5 hrs. Fig. **S5A, B and C**. Whole cell lysates were run on SDS-PAGE gels and immunoblotted using the indicated antibodies. GSK3 α/β activity is attenuated by activated AKT, and the inhibition of AKT kinase activity increases the activity of GSK3 α/β. Thus, the combination of GDC0941 and CT99021 was used to inhibit PI3K/AKT signalling while also inhibiting GSK3 α/β activity (N=1).

Supplementary Figure S6. Characterisation of the PINK1 antibody specificity.
SHSY5Y cells were transfected with either non-targeting scramble siRNA or a pool of four siRNA constructs targeting human PINK1 for 72 hrs. WB analysis of protein extracts from PINK1 and scramble knockdown cells. Actin is used as a loading control (N=1).

Supplementary Fig. S1

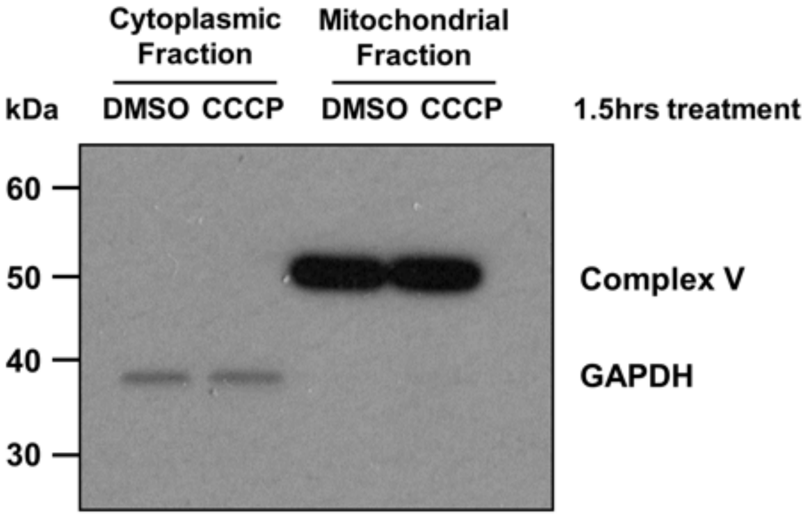

Supplementary Fig. S2

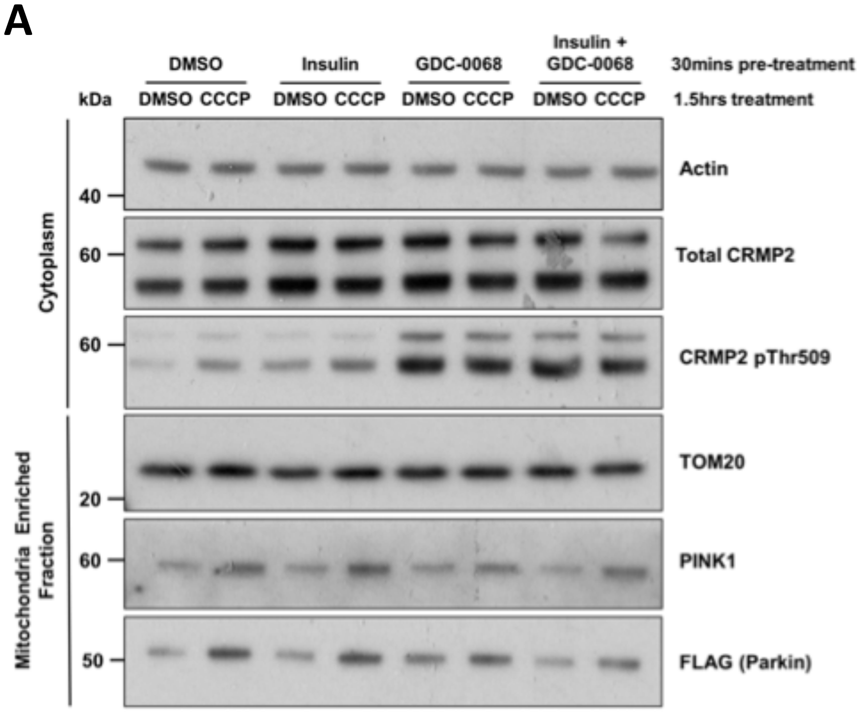

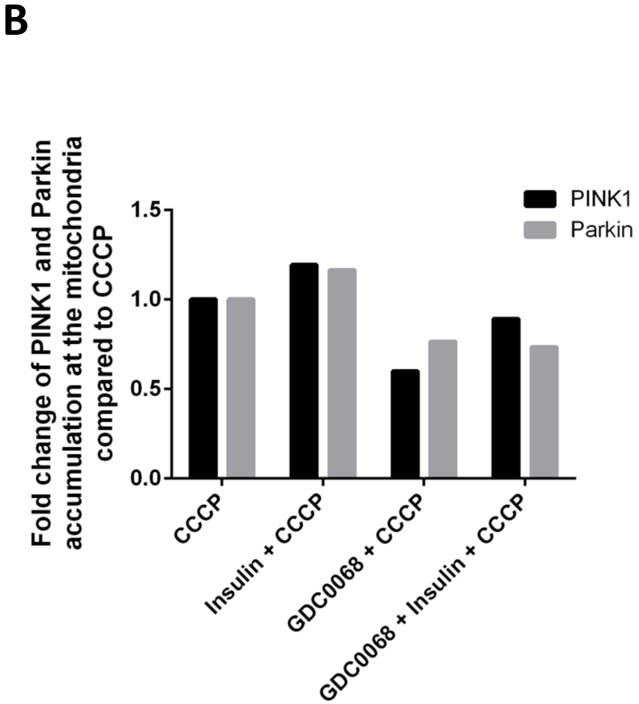

Supplementary Fig. S3

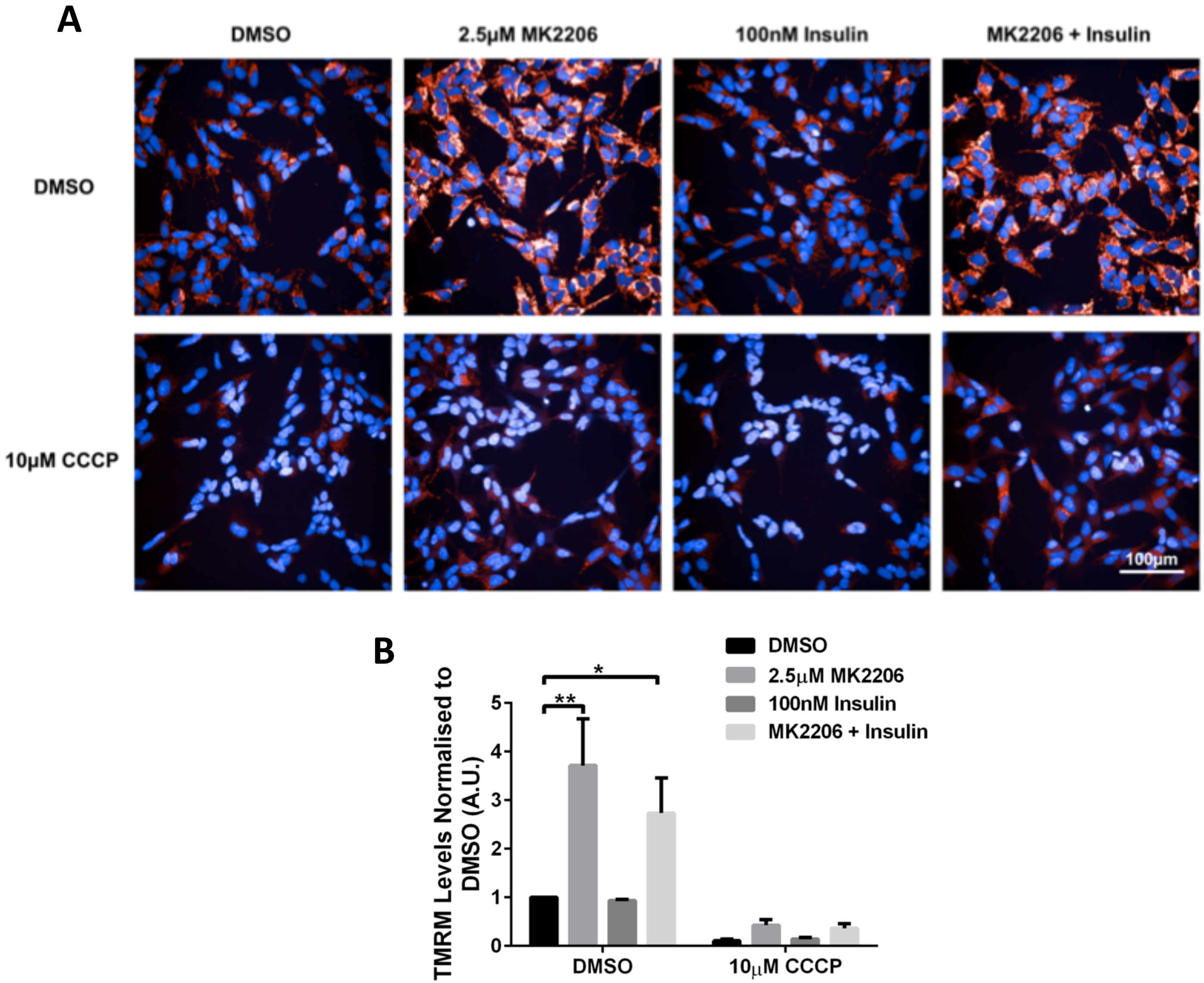

Supplementary Fig. S4

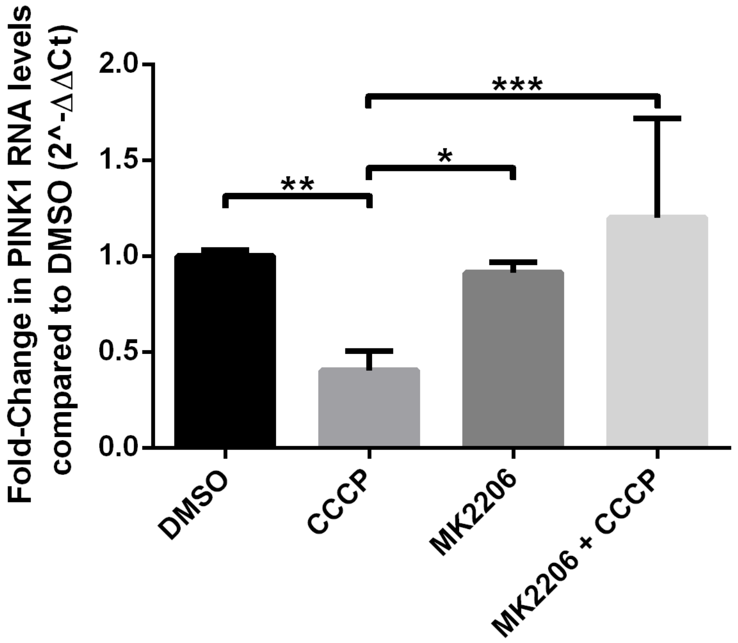

Supplementary Fig. S5

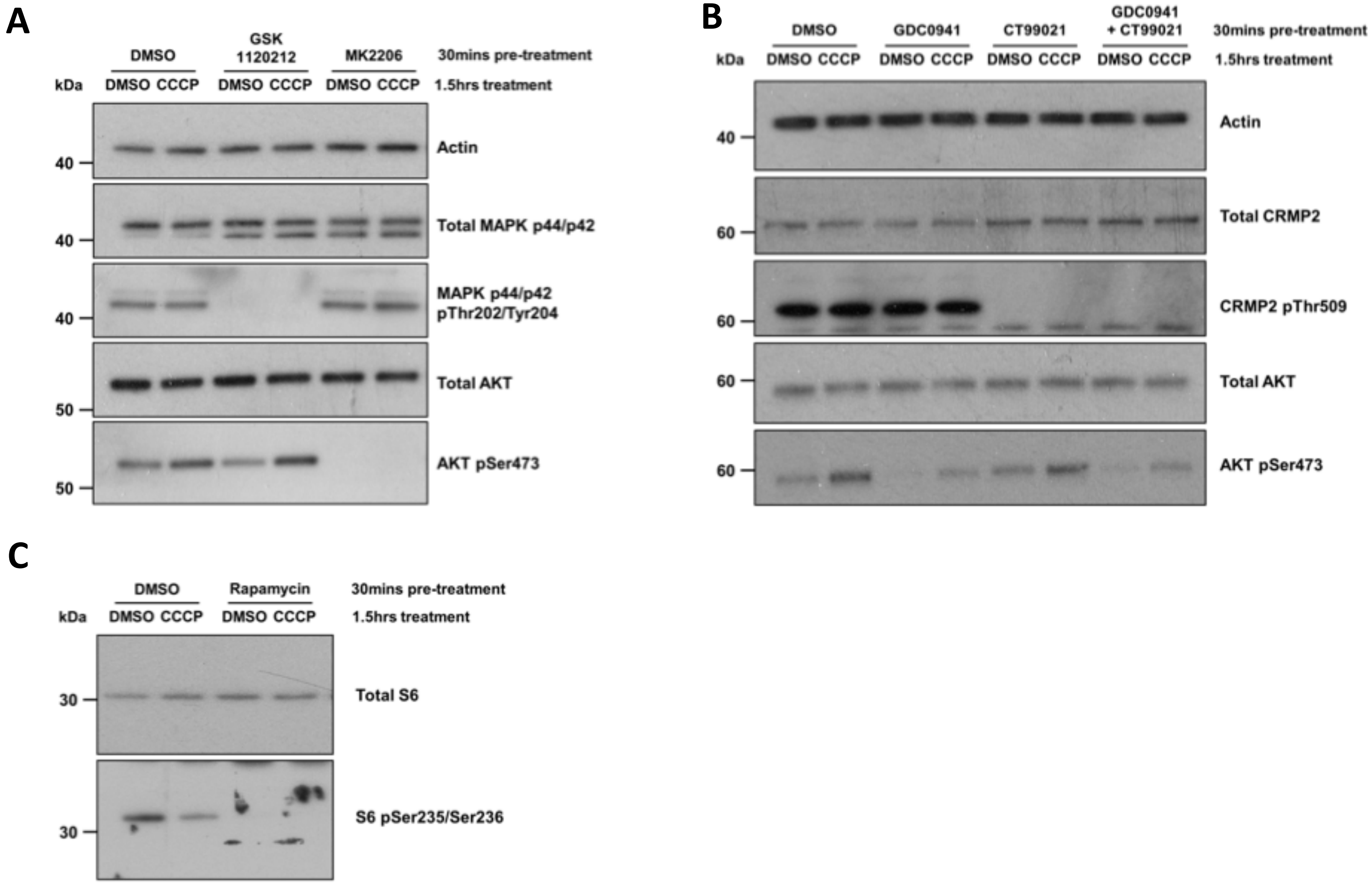

Supplementary Fig. S6

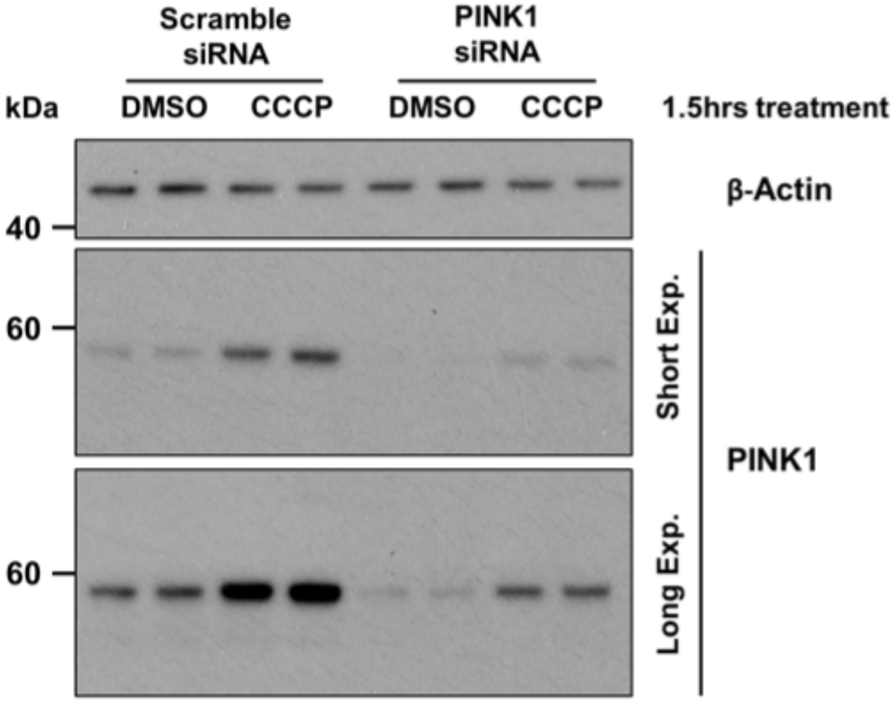

<u>Supplementary Data: - full-length blots</u>

FIG. 1A AKT signalling regulates PINK1 accumulation and Parkin recruitment to depolarised mitochondria.

FIG. 1A Total AKT Blot

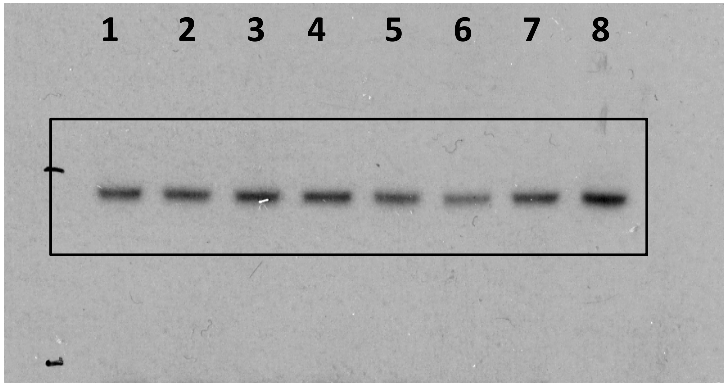

FIG. 1A AKT pSer473 Blot

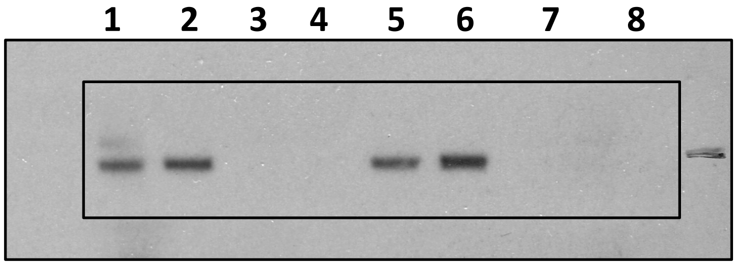

Sample	Treatment
1	DMSO
2	CCCP
3	MK2206
4	MK2206 CCCP
5	Insulin
6	Insuln CCCP
7	MK2206 Insulin
8	MK2206 Insulin CCCP

Boxed area used for figure

Supplementary Data: - full-length blots

FIG. 1C AKT signalling regulates PINK1 accumulation and Parkin recruitment to depolarised mitochondria.

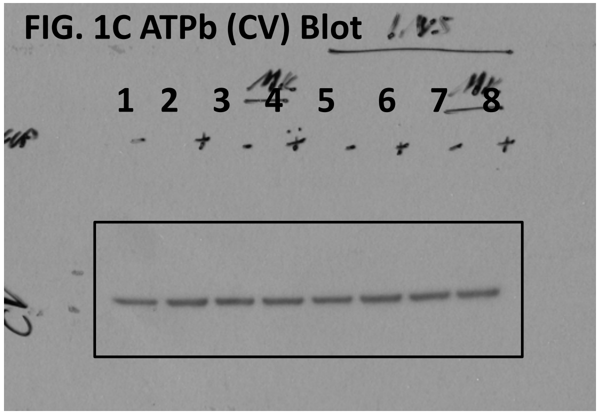

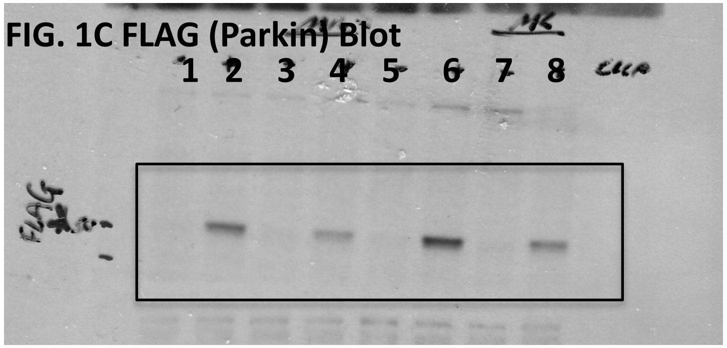

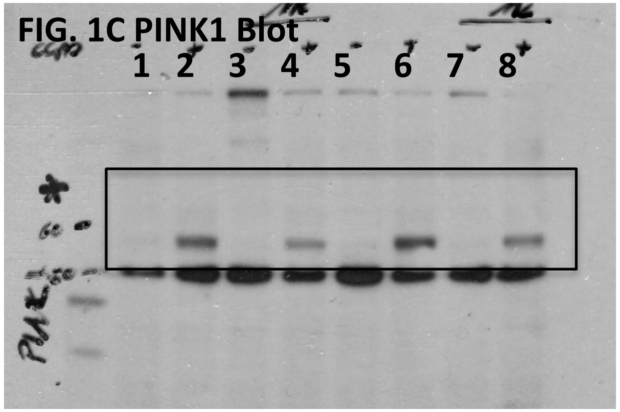

Sample	Treatment
1	DMSO
2	CCCP
3	MK2206
4	MK2206 CCCP
5	Insulin
6	Insuln CCCP
7	MK2206 Insulin
8	MK2206 Insulin CCCP

Boxed area used for figure

Supplementary Data: - full-length blots

FIG. 1F. AKT signalling regulates PINK1 accumulation and Parkin recruitment to depolarised mitochondria.

FIG. 1F Actin Blot

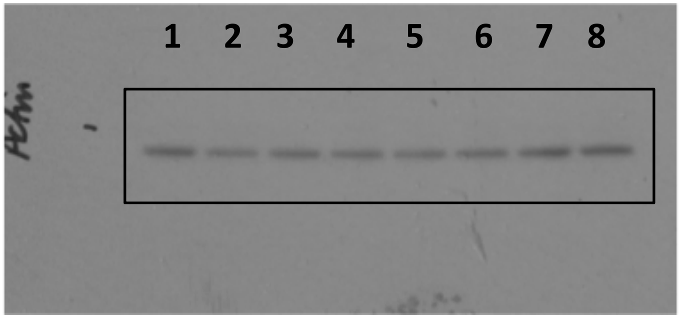

FIG. 1F ATPb (CV) Blot

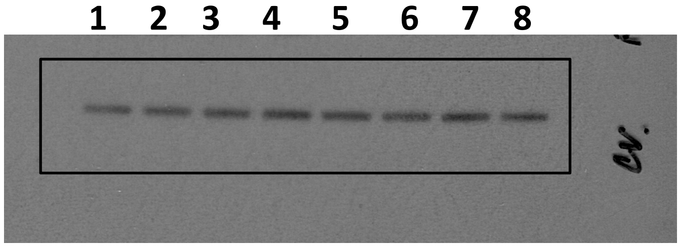

Sample	Treatment
1	DMSO
2	CCCP
3	MK2206
4	MK2206 CCCP
5	Insulin
6	Insuln CCCP
7	MK2206 Insulin
8	MK2206 Insulin CCCP

Boxed area used for figure

FIG. 1F Total AKT Blot

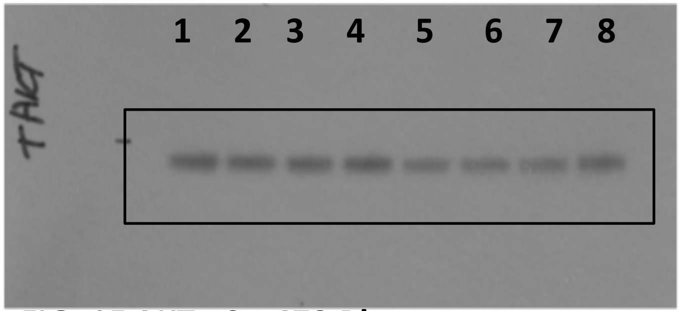

FIG. 1F PINK1 Blot

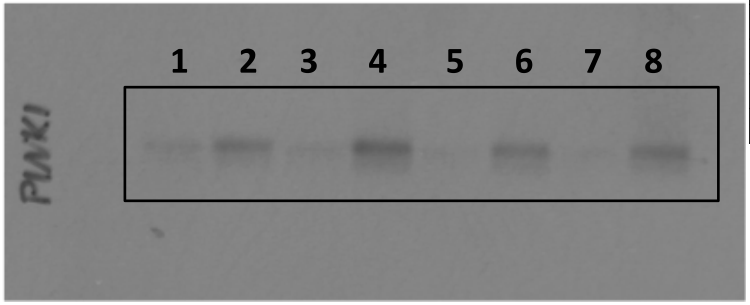

FIG. 1F AKT pSer473 Blot

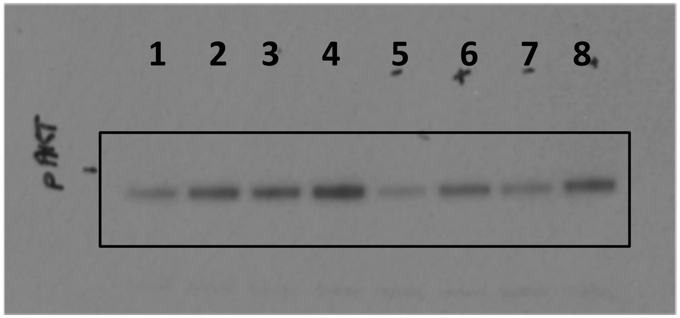

FIG. 1F FLAG (Parkin) Blot

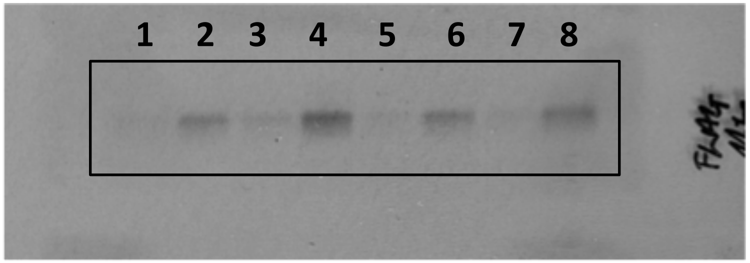

Supplementary Data: - full-length blots

Figure. 3E AKT signalling contributes to the regulation of mitophagy.

FIG. 3E Total AKT Blot

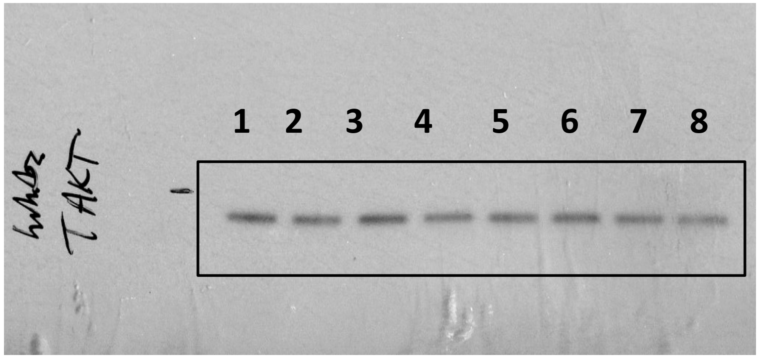

FIG. 3E AKT pSer473 Blot

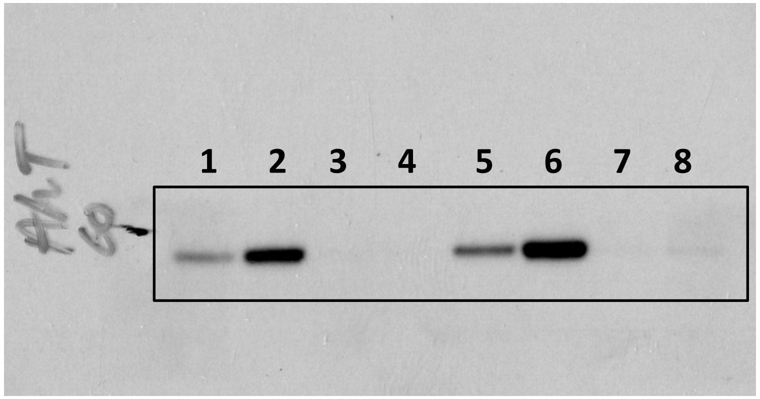

Sample	Treatment
1	DMSO
2	CCCP
3	MK2206
4	MK2206 CCCP
5	Insulin
6	Insuln CCCP
7	MK2206 Insulin
8	MK2206 Insulin CCCP

Boxed area used for figure

Supplementary Data: - full-length blots

Figure. 3G AKT signalling contributes to the regulation of mitophagy.

FIG. 3E Actin Blot

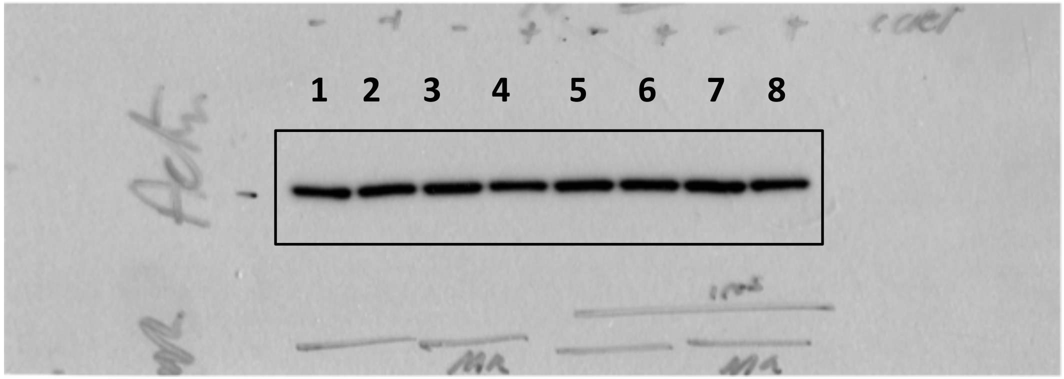

FIG. 3E TOM20 Blot

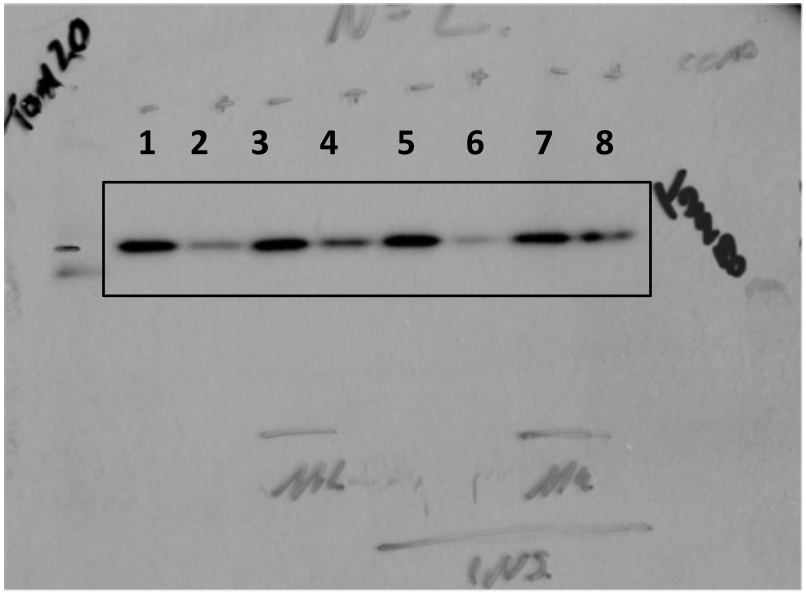

Sample	Treatment
1	DMSO
2	CCCP
3	MK2206
4	MK2206 CCCP
5	Insulin
6	Insuln CCCP
7	MK2206 Insulin
8	MK2206 Insulin CCCP

Boxed area used for figure

<u>Supplementary Data: - full-length blots</u>

Figure. 3I AKT signalling contributes to the regulation of mitophagy.

FIG. 3I Actin Blot

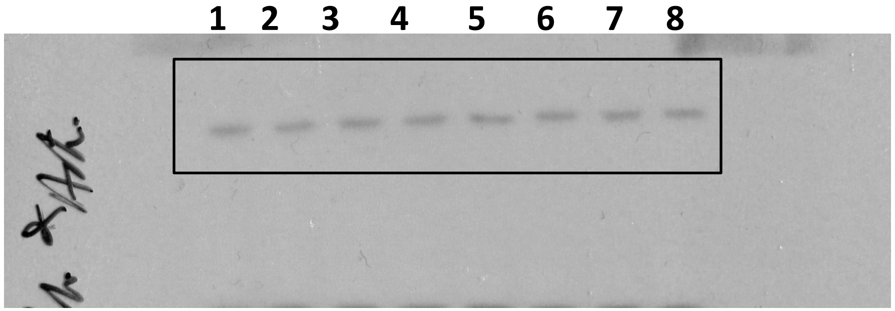

FIG. 3I ERAB Blot

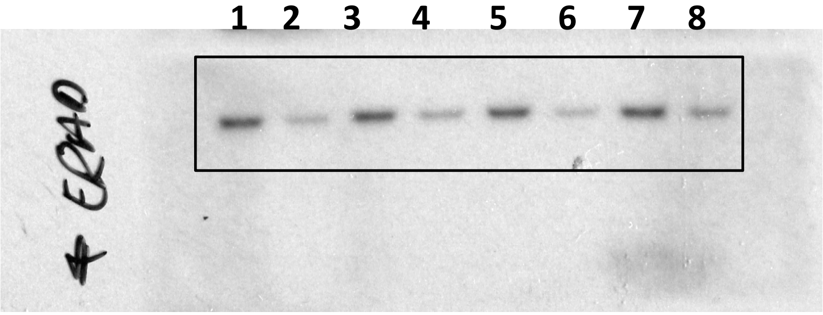

Sample	Treatment
1	DMSO
2	CCCP
3	MK2206
4	MK2206 CCCP
5	Insulin
6	Insuln CCCP
7	MK2206 Insulin
8	MK2206 Insulin CCCP

Boxed area used for figure

Supplementary Data: - full-length blots

Figure. 3K AKT signalling contributes to the regulation of mitophagy.

FIG. 3K GAPDH Blot

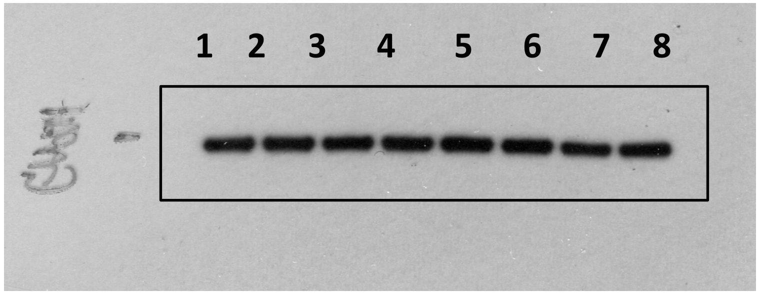

FIG. 3K Total AKT Blot

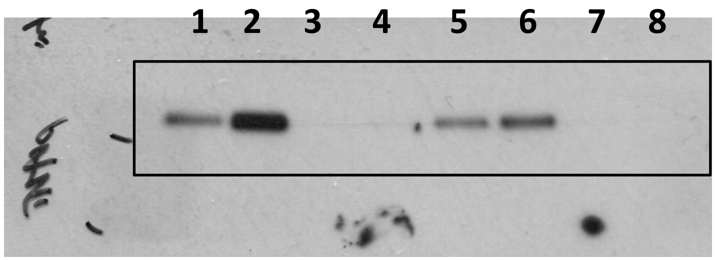

FIG. 3K Total S6 Blot

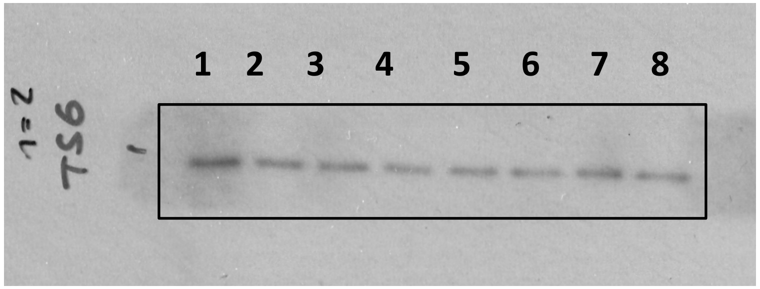

FIG. 3K pSer473 Blot

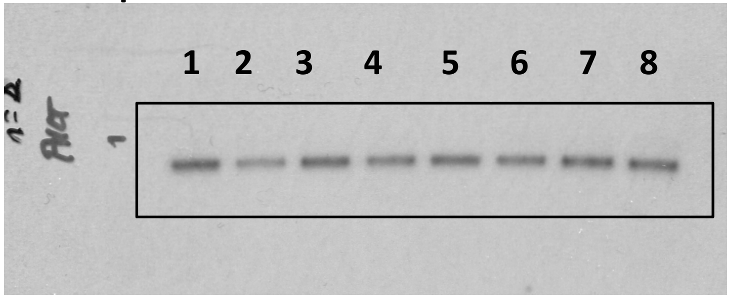

FIG. 3K S6 pSer235/236 Blot

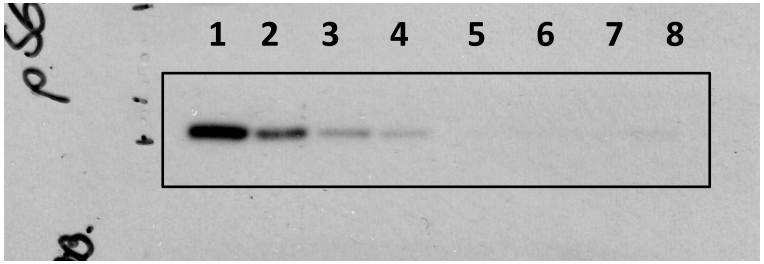

FIG. 3K ERAB Blot

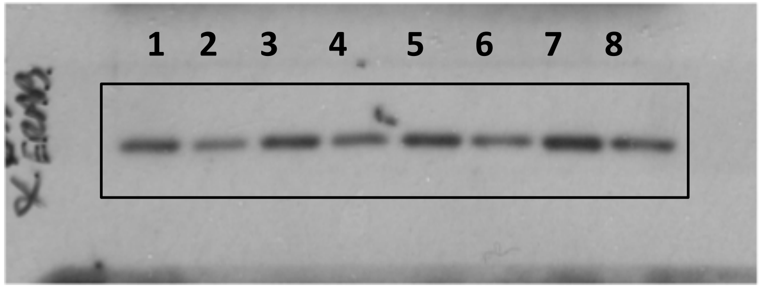

Sample	Treatment
1	DMSO
2	CCCP
3	MK2206
4	MK2206 CCCP

Boxed area used for figure

Supplementary Data: - full-length blots

FIG. 4A AKT kinase activity is required for mitophagy in iPSC cortical neurons.

FIG. 4A TOM20 Blot

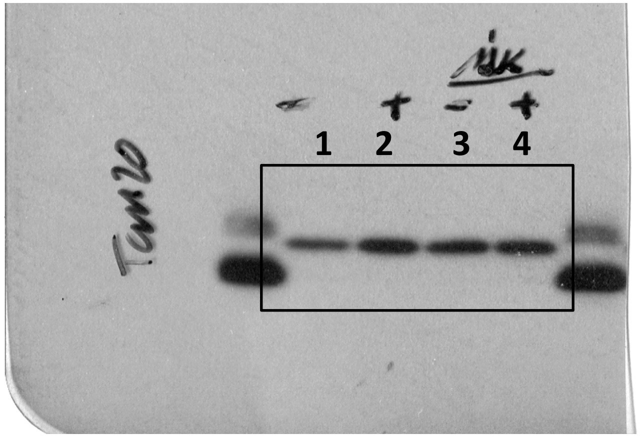

FIG. 4A PINK1 Blot

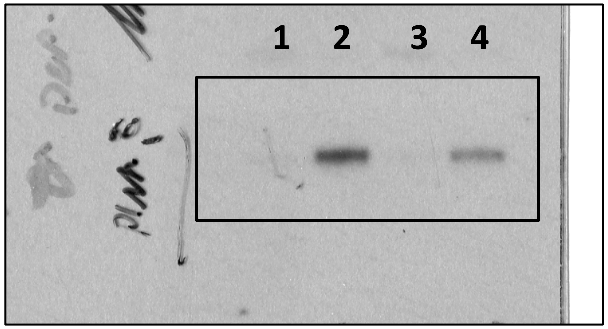

Sample	Treatment
1	DMSO
2	CCCP
3	MK2206
4	MK2206 CCCP

Boxed area used for figure

Supplementary Data: - full-length blots

FIG. 4C AKT kinase activity is required for mitophagy in iPSC cortical neurons.

FIG. 4C GAPDH blot

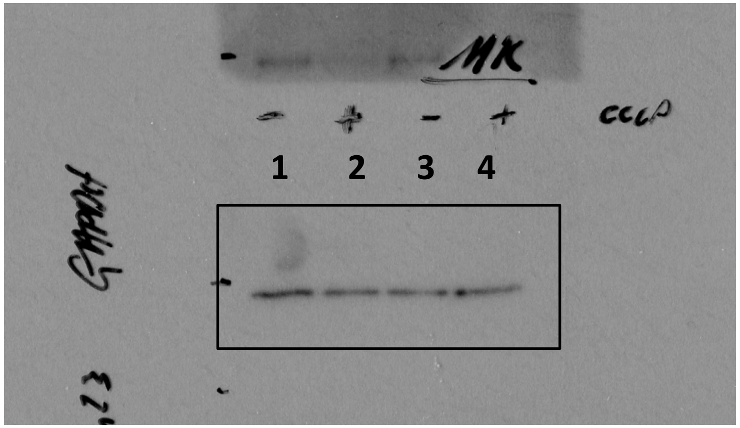

FIG. 4C AKT pSer473 blot

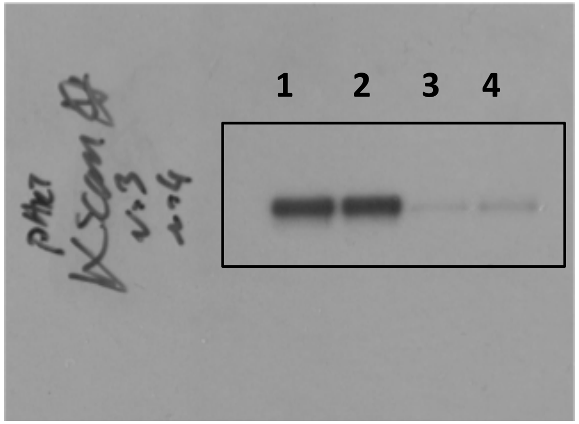

Sample	Treatment
1	DMSO
2	CCCP
3	MK2206
4	MK2206 CCCP

Boxed area used for figure

FIG. 4C TOM20 blot

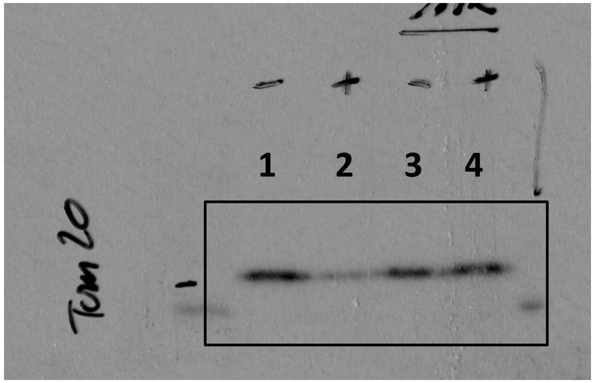

FIG. 4C Total AKT blot

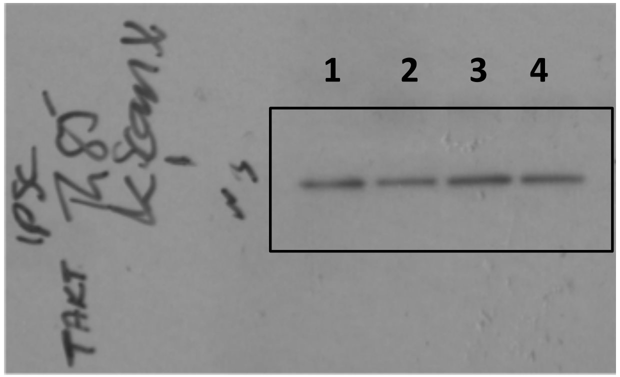

FIG. 4C TIM23 blot

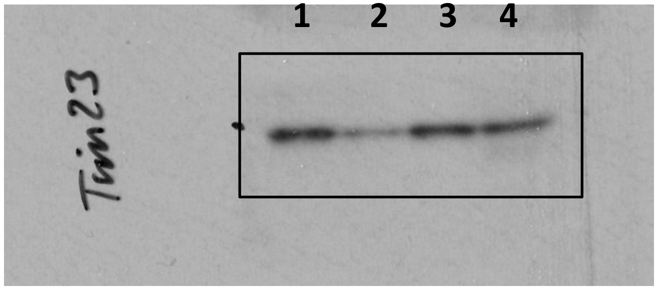

FIG. 4C PMPCB blot

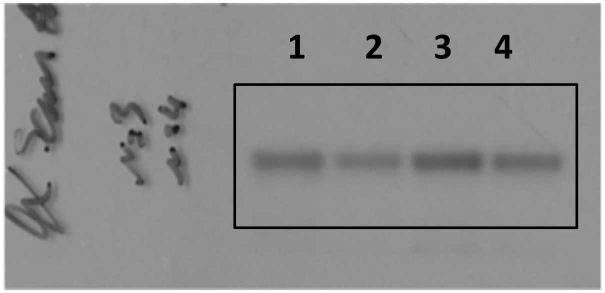

Supplement: Supplementary file 1 — Supplementary Information [file 41598_2018_26949_MOESM1_ESM.pdf]
